# Dosimetric analysis and clinical outcomes including adverse events in postoperative breast cancer irradiation involving the internal mammary lymph node region using Hybrid VMAT

**DOI:** 10.1007/s11604-025-01910-6

**Published:** 2025-11-20

**Authors:** Soichiro Ishihara, Yoshiko Doi, Minoru Nakao, Hideharu Miura, Shuichi Ozawa, Masahiro Kenjo

**Affiliations:** 1 Department of Radiation Oncology, Hiroshima Prefectural Hospital, 1-5-54, Ujinakanda, Minami-Ku, Hiroshima-Shi, Hiroshima, 734-8530 Japan; 2 Department of Radiation Oncology, Hiroshima High-Precision Radiotherapy Cancer Center, Hiroshima, Japan

**Keywords:** Breast cancer, Postoperative radiotherapy, Internal mammary lymph node, Volumetric modulated arc therapy, Dosimetric analysis

## Abstract

**Background:**

Although radiotherapy including the internal mammary lymph nodes (IMN) after breast surgery improves the prognosis of breast cancer, it may also increase radiation exposure to the heart and lungs due to the expanded irradiation field. Effectively and safely delivering radiation to the IMN region remains a clinical challenge.

**Purpose:**

At our institution, we originally developed a hybrid volumetric-modulated arc therapy (Hybrid VMAT) technique that combines conventional tangential fields with VMAT for postoperative breast cancer patients requiring irradiation to the conserved breast or chest wall and regional lymph nodes. In this study, we evaluated the safety, efficacy, and dosimetric performance of Hybrid VMAT in postoperative breast cancer patients whose treatment included the IMN region.

**Methods:**

This retrospective study included 57 patients who received Hybrid VMAT covering the chest wall or breast and regional lymph nodes, including the IMN region, between January 2016 and March 2023. Most patients (n = 53) underwent preoperative chemotherapy, and 44 had IMN metastases at diagnosis. The standard dose was 50 Gy in 25 fractions, with a 10 Gy boost in selected cases. Dosimetric parameters (Conformity index (CI), Homogeneity index (HI), and Organ at risk (OAR) doses), clinical outcomes (local/regional control, Progression-free survival (PFS), Overall survival (OS)), and adverse events (such as pneumonitis) were evaluated using CTCAE v5.0.

**Results:**

The median PTV CI and HI were 1.284 and 0.100, respectively, with a median IMN HI of 0.056. Mean heart and bilateral lung doses were kept low at 4.3 and 9.1 Gy, respectively. Median follow-up was 26 months. The 2 years local control, PFS, and OS rates were 96.3%, 79.2%, and 91.4%, respectively. No ≥ Grade 2 radiation pneumonitis occurred.

**Conclusion:**

This original Hybrid VMAT technique offers a clinically viable solution for balancing adequate IMN coverage and OAR protection, enabling safe combination with systemic therapies for breast cancer.

## Introduction

Radiation therapy (RT) plays a pivotal role in the local and regional management of breast cancer. It contributes to improved survival by reducing locoregional recurrence after both breast-conserving surgery and radical mastectomy [1, 2]. Regional nodal irradiation (RNI), including coverage of the supraclavicular and internal mammary lymph nodes (IMNs), is a specialized strategy for postoperative breast cancer patients [3, 4]. Its importance has been increasingly recognized in recent years [4].

However, adequate dose delivery to the IMNs remains technically challenging due to their deep anatomical location and proximity to critical organs such as the heart and lungs. To address these challenges, we previously developed an original irradiation technique, referred to as hybrid volumetric-modulated arc therapy (Hybrid VMAT), which combines VMAT with conventional tangential fields. This approach was designed to concentrate radiation dose to anatomically complex targets while minimizing exposure to surrounding organs at risk (OARs). Our earlier dosimetric study demonstrated that Hybrid VMAT provides superior dose distribution compared to conventional three-dimensional conformal radiotherapy (3D-CRT) [5].

Despite these advances, concerns persist regarding the increased risk of cardiopulmonary toxicity associated with postoperative irradiation that includes the IMNs. The Hybrid VMAT technique may offer a promising solution by enhancing dose conformity to the target while simultaneously reducing dose to OARs. Therefore, the aim of this study was to evaluate the dosimetric characteristics, clinical outcomes, and treatment-related toxicities of our institution’s original Hybrid VMAT technique in breast cancer patients undergoing postoperative RT that includes the IMNs.

## Materials and methods

### Patient data

This retrospective study included 57 patients who received Hybrid VMAT covering the chest wall or breast and regional lymph nodes, including the IMN region, between January 2016 and March 2023. Inclusion of the IMN region in the radiation field was based on either the presence of clinically apparent IMN metastasis at diagnosis—including cases with positive findings on positron emission tomography (PET)—or, in patients without obvious IMN involvement, on clinical judgment in situations such as an insufficient response to neoadjuvant chemotherapy or extensive axillary lymph node involvement observed during axillary dissection.

Prior to treatment, all patients provided written informed consent for the use of their clinical data in this study. The study protocol was approved by the institutional review board (approval number: E2022-0080).

### Contour definition

The clinical target volume (CTV) was defined as the entire ipsilateral chest area with supraclavicular nodes, axillary region and IMNs, based on the RTOG Breast Cancer Atlas and patient clinical data. When only sentinel lymph node biopsy was performed, the irradiated axillary field also included level I lymph nodes. Conversely, in cases with level I dissection, level I was generally excluded from the irradiation field. The IMNs range was adjusted for each patient to adequately include the metastatic areas. The planning target volume (PTV) was determined by adding a 5 mm margin to the CTV. Subsequently, a radiation oncologist manually modified the shape of the PTV. The PTV-mo (manually modified PTV) for evaluation and calculation was derived from PTV with a 2 mm margin on the skin surface and lungs surrounding the PTV. The OARs surrounding the targets, including the lungs, heart, spinal cord, and esophagus, were contoured. All delineation processes were reviewed by a radiation oncologist.

### Hybrid VMAT planning

**Fig. 1:**
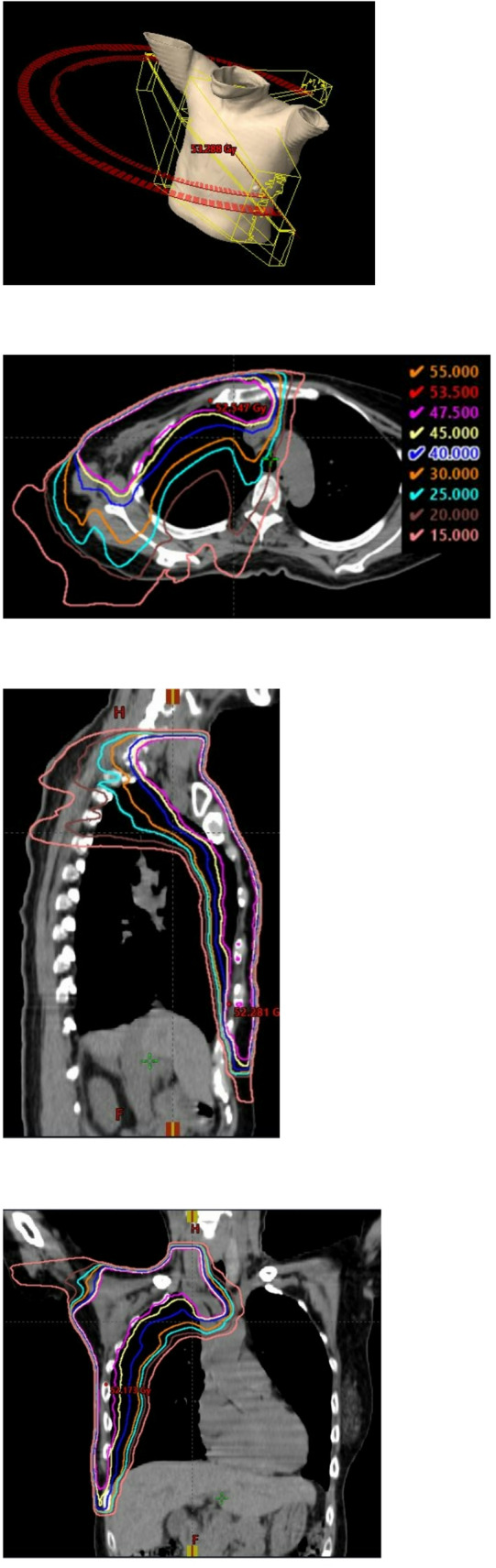
The beam arrangement and dose distibution of the Hybrid VMAT. **A** Overhead view of beam setup, and **B**–**D** distribution map. ‘Hybrid VMAT’ is combined conventional tangential field mainly for chest area and VMAT mainly for supraclavicular area and marginal zone

Figure 1 shows the beam arrangement of the Hybrid VMAT technique. Firstly, we set the isocenter point to the chest, which is 2 cm caudal to upper sternum, and calculated the main tangential fields for chest area. Subsequently, we optimized the VMAT plan for the supraclavicular, IMNs and chest areas based on the calculated results of the tangential fields for the chest area. The VMAT plan generated two coplanar arcs (one clockwise and another counterclockwise) with gantry rotation angles of 240° (ranging from 60° to 181° and 181° to 60° for right-sided primary tumours, and from 179° to 300° and 300° to 179° for left-sided primary tumours). The collimator angle of each arc was set to 10° or 80° to avoid tongue-and-groove effects. Details of the OAR and PTV dose constraints are presented in Table 1.

**Table 1:** Dose constraints for Hybrid-VMAT optimisation

		Constraint	(Preferable)
PTV-mo	D50%	97%–99%	–
	Maximum dose	≦115%	≦120%
	D95%	92%–96%	
Esophagus	D1cc	≦25 Gy	≦20 Gy
	D5cc	≦20 Gy	< ≦15 Gy
Lungs	V5Gy	≦35%	≦30%
	Mean dose	≦10 Gy	≦8 Gy

PTV-mo: manually modified planning target volume

All radiation plans were performed in the Eclipse treatment planning system (Varian Medical Systems, Palo Alto, CA, PRO, AcurosXB), using 6-MV photons generated by a linear accelerator (TrueBeam, Varian Medical Systems). Figure 1 shows the beam arrangement and dose distribution of the Hybrid VMAT.

To ensure accurate delivery of each Hybrid VMAT plan, daily bone matching was performed using orthogonal kV electronic portal images and digitally reconstructed radiographs created from planning computed tomography (CT) data. We also acquired kV cone-beam CT (CBCT) images after bone matching 2 or 3 days from the first delivery of a treatment and then weekly thereafter to verify patient position.

The prescription dose for all patients was 50 Gy in 25 fractions to the chest and regional lymph nodes, including the IMNs. In patients undergoing radical mastectomy, a 0.5 cm-thick solid bolus was added to the chest wall daily in the latter half of irradiation period to increase the skin dose. In breast-conserving surgery with close margins or in patients under 50 years, a 10 Gy boost in 5 fractions using 6–12 MeV electrons were applied to the tumor bed. For persistent metastatic lymph nodes or enlarged IMNs, which defined as radiologically abnormal nodes still evident on planning CT at the time of radiotherapy, an additional 10 Gy in 5 fractions was delivered at the clinician’s discretion.

### Plan evaluation

The results of 50 Gy in 25 fractions administered to the chest area and regional lymph nodes, including IMNs, were used to for physical verification of the treatment plan. In the dosimetric analysis, the following indices were extracted from the dose-volume histograms:

1) Homogeneity index (HI), and conformity index (CI) for PTV-mo and IMNs. The HI and CI were calculated according to the definition proposed by the International Commission on Radiation Units and Measurements [6] and expressed as follows:

$$\mathrm{HI}= \frac{\mathrm{D}2\mathrm{\%}- \mathrm{D}98\mathrm{\%}}{\mathrm{D}50\mathrm{\%}}\text{ and CI}= \frac{\mathrm{TV}\ge 95\mathrm{\%}}{\mathrm{PTV}\ge 95\mathrm{\%}}$$

where D2%, D98%, and D50% represent the doses covering 2%, 98%, and 50% of PTV-mo, respectively; TV ≥ 95% denotes the volume of the body that receives at least 95% of the prescribed dose, and PTV-mo ≥ 95% denotes the volume of PTV-mo that is covered by at least 95% of the prescribed dose.

2) Irradiation dose to OARs, such as lungs (the ipsilateral lung, contralateral lung, and bilateral lungs), heart, and esophagus.

### Follow-up and statistical methods

The follow-up period was defined as the number of days elapsed since the start of radiotherapy. Local and regional control, progression-free survival (PFS), and overall survival (OS) rates of the participants included in this study were calculated using Kaplan–Meier analysis using the EZR (Saitama Medical Center, Jichi Medical University), a graphical user interface for R (The R Foundation Computing, version 3.4.1).

For the evaluation of adverse events (AEs), each patient was monitored by their physician twice weekly during radiotherapy and at several-month intervals after the completion of radiotherapy for up to one year. The timing and location of the most severe radiation dermatitis in each patient were recorded. Radiation pneumonitis was assessed within 6 months to 1 year after radiotherapy. All adverse reactions were graded according to the Common Terminology Criteria for Adverse Events (CTCAE), Version 5.0, published by the National Cancer Institute and were reported as the worst grade observed during the follow-up period.

## Results

### Clinical characteristics

A total of 57 patients were included in this study. Their clinical characteristics are summarized in Table 2. The median age was 52 years (range: 26–79), and 56 patients (98.2%) were female. Right-sided tumors were present in 24 patients and left-sided tumors in 33. At initial diagnosis, IMN metastasis was confirmed in 44 patients (77.2%). Forty-six patients (80.7%) underwent total mastectomy, while 11 patients underwent breast-conserving surgery. Axillary dissection was performed in 50 patients, and the remaining 7 underwent sentinel lymph node biopsy. In these cases, sentinel lymph node biopsy was selected by the referring surgeons after favorable responses to systemic chemotherapy. Of the 50 patients who underwent axillary dissection, 30 underwent level I dissection, 19 underwent levels I and II dissection, and 1 underwent levels I, II, and III dissection, respectively.

**Table 2:** Patient characteristics

Characteristics and treatment	Overall
patients	57
Median age (range), years	52(26–79)
Sex: Female/Male	56/1
Laterality: Right/Left	24/33
cT factor: 1/2/3/4	2/27/14/14
cN stage: 0/1/2/3	2/2/9/44
cM stage: 0/1	55/2
IMN metastasis: Yes/No	44/13
Mastectomy: Total/Partial	46/11
Axillary treatment: axillary dissection/sentinel lymph node biopsy	50/7
Axillary dissection site: only I/I + II/I + II + III	30 /19/1
Neoadjuvant chemotherapy: Yes/No	53/4
Histological therapeutic effect: un-known/G1a/G1b/G2a/G2b/G3	7/10/10/12/3/15
Cases with additional boost radiotherapy (10 Gy/5fr)	29
Tumor bed boost	11*
residual lymph node metastasis	4*
IMN	23*

^*^Duplicate counts

IMN: Internal mammary node

Neoadjuvant chemotherapy was administered to 53 patients (93.0%). Neoadjuvant chemotherapy primarily consisted of taxanes and anthracyclines. Based on the discretion of the attending physicians, some patients received dose-dense regimens or the addition of bevacizumab to standard chemotherapy. In HER2-positive cases, anti-HER2 monoclonal antibodies were administered. Regarding the pathological response to chemotherapy, seven patients had unknown response grades, and the remaining showed the following distribution: G1a in 10, G1b in 10, G2a in 12, G2b in 3, and G3 in 15. For maintenance therapy, patients with hormone receptor–positive breast cancer received endocrine treatment, while those with HER2-positive disease continued anti-HER2 antibody therapy for the scheduled duration. Since 2018, cyclin-dependent kinase 4 and 6 (CDK4/6) inhibitors approved for use as maintenance chemotherapy in Japan have been administered to eligible patients.

Boost irradiation with 10 Gy in 5 fractions was delivered in 29 patients. Among these, 11 patients received boosts to the tumor bed, 4 to residual lymph nodes, and 23 to the IMNs. Some patients received boosts to multiple sites. The indication for IMN boost irradiation was the presence of residual IMNs observed on imaging at the time of radiotherapy planning.

### Evaluation of dosimetric parameters

**Fig. 2:**
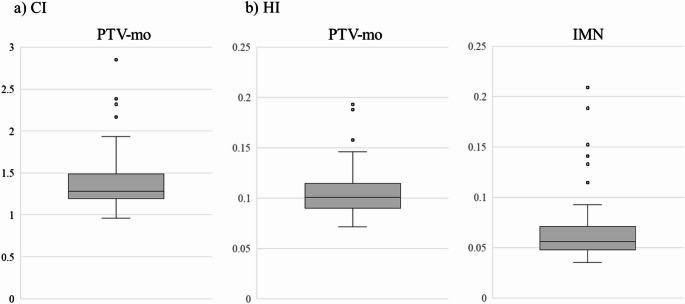
The box-and-whisker plot for **A** CI of PTV-mo, and **B** HI of PTV-mo and IMN, respectively. The band inside the boxes represents the median, and the bottom and top of the boxes represent the 25th and 75th percentiles, respectively. The whiskers indicate the lowest datum still within the 1.5 interquartile range (IQR) of the lower quartile, and the highest datum still within the 1.5 IQR of the upper quartile. Dots (‘o’) indicate outliners of maximum displacement. The CI for PTV-mo was median 1.284 (range: 0.959–2.849), and the HI for PTV-mo median 0.101 (range: 0.072–0.193). The HI for IMNs was median 0.056 (range: 0.035–0.209)

The CI and HI of the PTV-mo and the IMNs are illustrated in Fig. 2. For the PTV-mo, the median CI was 1.284 (range: 0.959–2.849), and the median HI was 0.101 (range: 0.072–0.193). For the IMNs, the median HI was 0.056 (range: 0.035–0.209). These consistently low HI values indicate that the prescribed dose was delivered to PTV-mo and the IMNs with good uniformity.

The OAR dose parameters are presented in Table 3. The mean dose to the ipsilateral lung was 16.3 Gy (range: 1.4–23.2 Gy), with a V20Gy of 33.8% and V15Gy of 39.2%. The mean dose to the contralateral lung was 2.2 Gy (range: 0.95–17.7 Gy), with a V5Gy of 6.7%. For both lungs combined, the mean dose was 9.11 Gy (range: 6.60–14.8 Gy), with a V20Gy of 16.7% and V5Gy of 32.6%. The mean dose to the heart was 4.30 Gy (range: 1.05–13.7 Gy), with 5.45 Gy for left-sided tumors and 2.82 Gy for right-sided tumors. The D1cc and D5cc values for the esophagus were 17.4 and 12.8 Gy, respectively.

**Table 3:** Evaluation of toxicity

OARs	Mean parameters for all plans (range, SD)
Ipsilateral lung	
Mean dose	16.3 Gy (1.4 -23.2, SD: 3.20)
V20Gy	33.8% (0–49.8, SD: 7.23)
V15Gy	39.2% (0–54.9, SD: 7.88)
Contralateral lung	
Mean dose	2.2 Gy (0.95–17.7, SD: 2.19)
V5Gy	6.67% (0.48–61.2, SD: 9.68)
Bilateral lungs	
Mean dose	9.11 Gy (6.60–14.8, SD: 1.36)
V20Gy	16.7% (10.4–28.8, SD: 3.12)
V5Gy	32.6% (22.0–54.0, SD: 6.42)
Esophagus	
D1cc	17.4 Gy (10.8–28.9, SD: 4.28)
D5cc	12.8 Gy (3.38–21.0, SD: 3.41)
Heart	
Mean dose	4.30 Gy (1.05 Gy -13.7 Gy, SD: 2.92 Gy) (Lt: n = 33) 5.45 Gy (2.33–13.7, SD: 2.91) (Rt: n = 24) 2.82 Gy (1.05 -7.23, SD: 1.70)

### Evaluation of clinical outcomes and adverse events

**Fig. 3:**
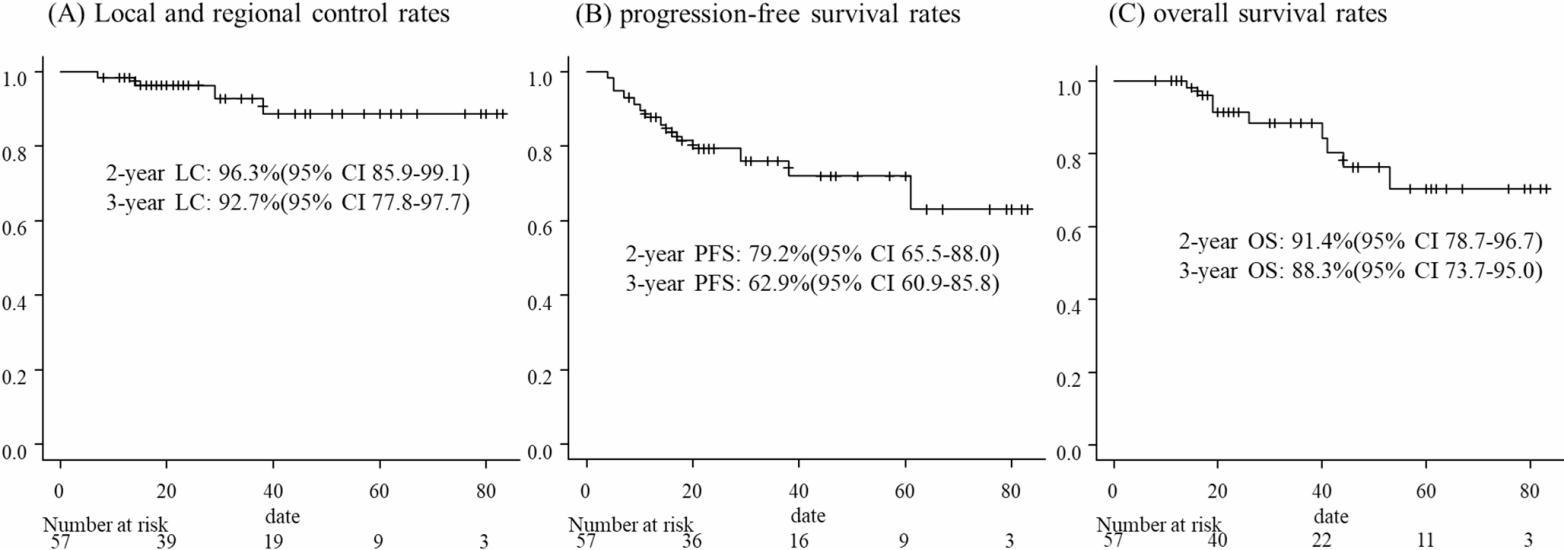
Treatment results of Hybrid VMAT for chest area and regional node included IMN. Median follow-up period was 26 months (range: 8–83). **A** Local and regional control rates. The 2- and 3 year OS rates were 96.3% (95% confidence interval [CI], 85.9%–99.1%) and 92.7% (95% CI 77.8%–97.7%), respectively. **B** progression-free survival (PFS) rates. The 2- and 3 years PFS rates were 79.2% (95% CI 65.5%–88.0%) and 62.9% (95% CI 60.9%–85.8%), respectively. **C** overall survival rates (OS) rates. The 2- and 3 years OS rates were 91.4% (95% CI 78.7%–96.7%) and 88.3% (95% CI 73.7%–95.0%), respectively

The median follow-up period was 26 months (range: 8–83). The 2- and 3-year local and regional control rates were 96.3% (95% confidence interval [CI] 85.9–99.1%) and 92.7% (95% CI 77.8–97.7%), respectively (Fig. 3A). The 2- and 3-year PFS rates were 79.2% (95% CI 65.5–88.0%) and 62.9% (95% CI 60.9–85.8%), respectively (Fig. 3B). The 2- and 3-year OS rates were 91.4% (95% CI 78.7–96.7%) and 88.3% (95% CI 73.7–95.0%), respectively (Fig. 3C).

**Fig. 4:**
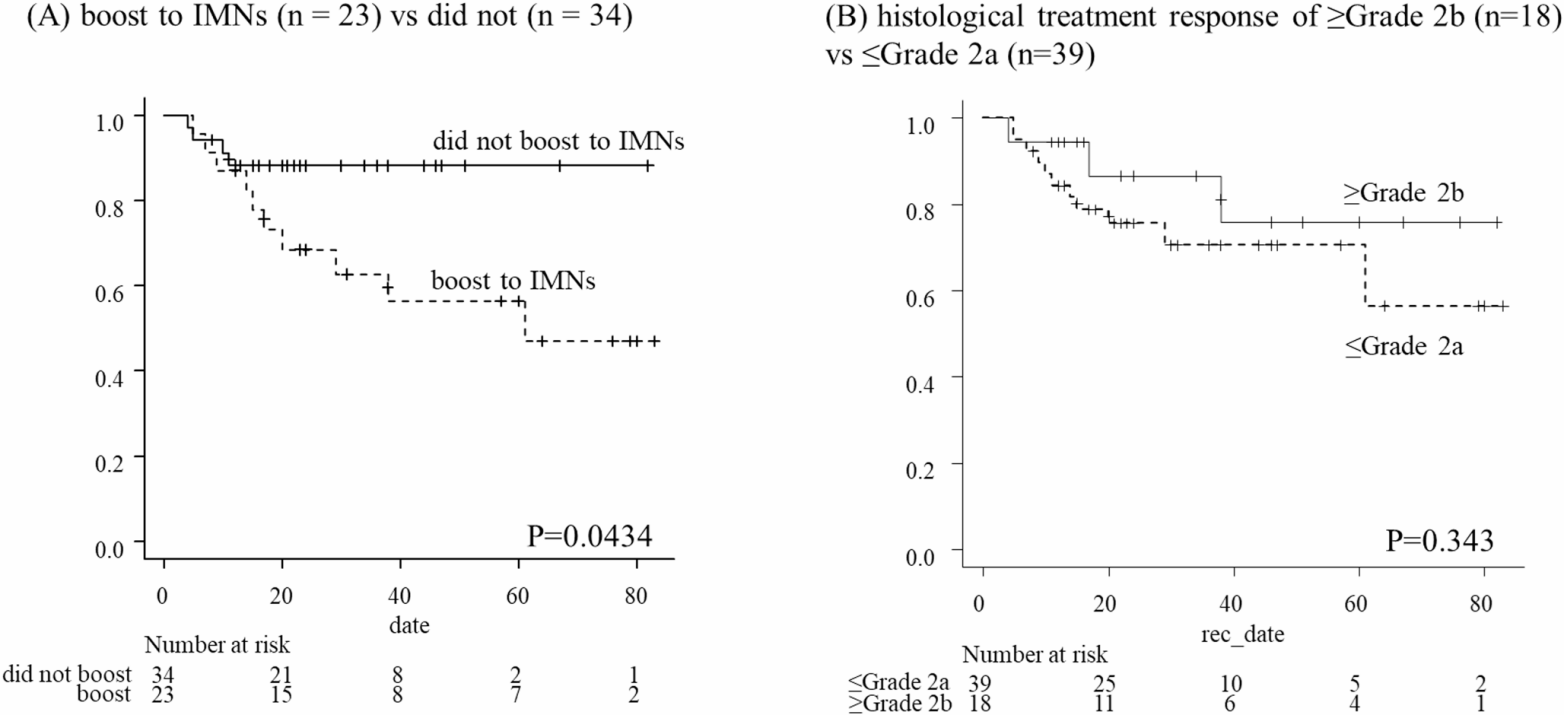
The progression-free survival rate. **A** received a boost to IMNs (n = 23): dotted line, with those who did not (n = 34): direct line. The 2 year PFS rates were 68.3% (95% confidence interval [CI] 44.7–83.5%) in the subgroup of boost to IMNs vs 88.0% (95% CI, 71.2–95.3%) in the subgroup of did not boost to IMNs, *p* = 0.0434, respectively. **B** patients with a histological treatment response of ≥ Grade 2b (n = 18) and those with ≤ Grade 2a (n = 39). The 2 years PFS rates were 86.6% (95% CI, 55.2–96.6%) in the subgroup of ≥ Grade 2b vs 75.6% (95% CI 58.2–86.6%) in the subgroup of ≤ Grade 2b, *p* = 0.343, respectively

In the subgroup analysis, the 2-year PFS was significantly lower in patients who received an IMN boost (n = 23) compared to those who did not (n = 34), with rates of 68.3% (95% CI 44.7–83.5%) and 88.0% (95% CI 71.2–95.3%), respectively (p = 0.043) (Fig. 4A). All recurrences in the IMN boost group were associated with concurrent distant metastases.

 Furthermore, patients were stratified according to their pathological response to neoadjuvant chemotherapy. Patients with a response of Grade 2b or higher (n = 18) showed a trend toward better 2 years PFS (86.6%, 95% CI 55.2–96.6%) compared to those with Grade 2a or lower (n = 39; 75.6%, 95% CI 58.2–86.6%), although the difference was not statistically significant (*p* = 0.343) (Fig. 4B).

Adverse events are summarized in Table 4. Radiation dermatitis was observed in 98.2% of patients, with Grade 1, Grade 2, and Grade 3 events occurring in 52.6%, 45.6%, and 1.8%, respectively. Radiation esophagitis was observed in 22.8% of patients, all of which were Grade 1. Radiation pneumonitis was observed in 5.3% of patients, also limited to Grade 1. Upper limb lymphedema occurred in 3.5% of patients at Grade 1 and 1.8% at Grade 2. No Grade 4 or higher adverse events were observed.

**Table 4:** Adverse events (CTCAE v5.0)

	Grade 0	Grade1	Grade2	Grade3
Radiation dermatitis	0 (0%)	30 (52.6%)	26 (45.6%)	1 (1.8%)
Radiation esophagitis	44 (77.2%)	13 (22.8%)	0 (0%)	0 (0%)
Radiation pneumonitis	54 (94.7%)	3 (5.3%)	0 (0%)	0 (0%)
Secondary upper extremity edema	54 (94.7%)	2 (3.5%)	1 (1.8%)	0

## Discussion

The postoperative radiotherapy for breast cancer tends to increase radiation exposure to surrounding normal tissues such as the lungs and heart, especially when the IMNs are included in the clinical target volume. In this study, the use of Hybrid VMAT enabled adequate dose coverage of the target volume while effectively minimizing radiation exposure to adjacent organs, even when IMNs were irradiated. A notable feature of Hybrid VMAT is its incorporation of tangential fields, which distinguishes it from conventional VMAT and contributes to dose sparing of the contralateral lung. Furthermore, evaluation using the HI confirmed that uniform and sufficient dose delivery to the IMN region was consistently achieved, supporting the robustness of this technique. Accordingly, in our series, IMN boost irradiation was not required due to insufficient IMN dose coverage, but rather was selectively applied in cases with residual or enlarged IMN disease after systemic therapy. In most cases where IMRT is applied to PMRT, the entire treatment is delivered using VMAT. Yano et al. reported the dosimetric outcomes of full-arc VMAT for PMRT including IMN irradiation, with a mean dose to both lungs of 8.08 Gy and a lung V5Gy of 38.44% [7]. In comparison, our Hybrid VMAT approach achieved a slightly higher mean lung dose of 9.11 Gy but a lower V5Gy of 32.6%. These findings suggest that the Hybrid VMAT technique, by incorporating tangential beams, effectively reduces the low-dose irradiated volume without significantly increasing the high-dose area, which may offer a favorable balance in clinical practice.

However, combining tangential irradiation with VMAT requires caution due to the potential for hot or cold spots at the junction of the two techniques. To address this issue, we carefully optimized the dose gradient at the interface between the tangential and VMAT fields in the thoracic region. Miura et al. have demonstrated that this approach improves the robustness of treatment plans [8]. Moreover, the open-field design of the tangential component in Hybrid VMAT is less sensitive to anterior–posterior respiratory motion, enhancing both reproducibility and target coverage compared with VMAT alone. Consequently, the Hybrid VMAT technique developed at our institution may offer superior robustness against daily setup variability.

In our cohort, 44 patients (77%) presented with IMN metastases at diagnosis. For patients with breast cancer and positive IMNs, including the IMNs in the PMRT field is critical for effective locoregional control. A previous multicenter retrospective study by Kim et al. reported 5 years PFS and OS rates of 68.6 and 81.8%, respectively, in patients with IMN-positive breast cancer treated with NAC followed by postoperative radiotherapy including IMNs [9]. In comparison, our study demonstrated 3-year PFS and OS rates of 62.9 and 88.3%, respectively. Although the median follow-up period in our study was relatively short (26 months), these outcomes appear comparable.

Considering the generally poor prognosis of IMN-positive disease, treatment intensification is an important consideration. Yang et al. stratified patients according to the radiation dose to the IMNs and reported that high-dose irradiation (63.6–70.4 Gy) was associated with improved PFS in patients with IMNs ≥ 1 cm [10]. However, our findings contradicted theirs. In our study, a boost to the IMNs was administered when residual enlarged IMN-positive lymph nodes were observed on planning CT at the time of treatment simulation. These cases represented patients with persistent IMN disease after systemic therapy, but not necessarily progressive or definitively chemotherapy-resistant disease. Interestingly, patients who received the IMN boost had worse PFS than those who did not (62.6 vs. 88.0%, *p* = 0.043), and all recurrences in the boost group were associated with distant metastases. These results suggest that high-dose irradiation intended for local control of IMN metastases did not translate into improved prognosis. Given that the majority of patients in this study received neoadjuvant chemotherapy, the presence of residual IMN disease at the time of radiotherapy planning may serve as a prognostic indicator of poor systemic control. Furthermore, although not statistically significant, a trend toward improved PFS was observed in patients who achieved a pathological response of Grade 2b or higher following neoadjuvant chemotherapy. Taken together, these findings suggest that, in IMN-positive breast cancer, intensifying systemic therapy may be more beneficial for improving PFS than increasing the radiation dose.

Recently, the results of the NSABP B-51 trial demonstrated that omitting postoperative radiotherapy in patients with clinically node-positive breast cancer (cN +) who convert to pathological node-negative (ypN0) status after neoadjuvant chemotherapy does not compromise treatment outcomes [11]. Currently, in our institution, all patients with confirmed IMN-positive disease, as well as selected high-risk patients without confirmed IMN metastasis (e.g., inner or central tumor location, extensive axillary involvement, or insufficient response to neoadjuvant chemotherapy), receive irradiation that includes the IMN region. However, in the future, it may become feasible to tailor both the irradiation field and radiation dose based on the response to neoadjuvant chemotherapy.

Radiotherapy involving the IMNs is also associated with an increased risk of radiation pneumonitis due to higher lung dose. In prior randomized trials including IMN irradiation, such as the MA.20 [12] and KROG 08–06 [13] studies, Grade 2 RP was observed in 1.2% and 2.5% of patients, respectively. In our study, no patients experienced ≥ Grade 2 radiation pneumonitis, indicating the feasibility and safety of our treatment technique. This low incidence of radiation pneumonitis may be attributed to the high precision of Hybrid VMAT, which effectively spares lung tissue. Because IMN-positive breast cancer patients often require continued systemic therapy, including CDK4/6 inhibitors or trastuzumab emtansine (T-DM1)—both of which carry a risk of interstitial pneumonitis—minimizing pulmonary toxicity is crucial to ensure uninterrupted systemic treatment.

This study has several limitations. It was retrospective in nature, had a small sample size, and a relatively short follow-up period. These factors may limit the reliability of long-term outcome and toxicity assessments. Furthermore, prior reports have raised concerns that increased low-dose radiation exposure to organs at risk may elevate the risk of secondary malignancies [14]. Therefore, prospective studies with long-term follow-up are warranted to validate the safety and effectiveness of Hybrid VMAT in clinical practice.

## Conclusion

This study demonstrated that postoperative radiotherapy for breast cancer involving IMN irradiation using the Hybrid VMAT technique provided favorable dose distribution, reduced radiation exposure to surrounding normal tissues, and a low incidence of adverse events. Given its ability to maintain effective locoregional control while minimizing toxicity, Hybrid VMAT represents a promising strategy that supports the uninterrupted continuation of systemic therapy in breast cancer patients, particularly those requiring IMN coverage.
